# 3D-Printed Models as Catalysts for Neuroanatomy Learning: An Immersive Educational Experience for Medical Students

**DOI:** 10.7759/cureus.116046

**Published:** 2026-09-10

**Authors:** Noé Pérez Carrillo, Dan López Vázquez, Erick David Muñoz Carpio, Mixel Zair Baltierra Hernández, Francisco de Jesús García-Mendoza

**Affiliations:** 1 Neurosurgery, General Hospital of Mexico "Dr. Eduardo Liceaga", Mexico City, MEX; 2 Neurosurgery, Hospital de Jesus, Mexico City, MEX; 3 Faculty of Medicine, Universidad Autónoma Metropolitana Unidad Xochimilco, Mexico City, MEX; 4 Central Research Laboratory, Faculty of Medicine, Benemérita Universidad Autónoma de Puebla, Puebla, MEX

**Keywords:** anatomical models, educational innovation, medical education, neuroanatomy, three-dimensional printing

## Abstract

Introduction

Neuroanatomy presents unique challenges due to the three-dimensional (3D) complexity of the nervous system. This study evaluates the impact of 3D-printed brainstem models on neuroanatomy teaching to medical students.

Methods

A descriptive, cross-sectional, non-randomized, between-group study was conducted to compare students’ perceptions of 3D-printed anatomical models versus traditional schematic diagrams. Participants were recruited through non-probability convenience sampling and allocated to a 3D-model group (n = 38) or a traditional-diagram group (n = 23). A Likert-type questionnaire assessed perceived motivation, clarity, understanding, usefulness, and satisfaction. Descriptive statistics and the Mann-Whitney U test were used for between-group comparisons.

Results

Sixty-one responses were analyzed. Compared with traditional schematic diagrams, 3D-printed models received significantly higher ratings for conceptual clarity, overall satisfaction, and preference for future use (p < 0.05). In contrast, schematic diagrams received higher ratings for motivation, a finding that may reflect unmeasured factors such as prior familiarity with conventional learning resources.

Conclusions

The use of 3D printing improves the neuroanatomical learning experience for medical students, as it is perceived as an innovative, clear, and complementary resource. Its implementation can transform traditional neuroscience teaching.

## Introduction

The teaching of anatomy, an essential pillar in the first years of medical training, has faced multiple methodological challenges, especially in areas such as neuroanatomy, where the complex and three-dimensional (3D) structures of the central nervous system require advanced teaching tools for their proper understanding [1,2]. Traditional methods such as dissection, synthetic models, anatomical atlases, and illustrated diagrams remain of great value, but they have significant limitations, for example, the deterioration of cadaveric material, the restricted availability of specimens, high costs, and the limited spatial perception offered by two-dimensional resources [3-5].

Three-dimensional printing has emerged as an educational tool that enables the creation of tangible, realistic, and reproducible anatomical models based on clinical data such as CT scans or MRIs. This technology has been shown to improve academic performance, anatomical understanding, motivation, and student satisfaction, especially in settings where access to cadavers is limited [6-8]. In addition, it offers advantages such as customization, relatively low cost, portability, and utility in clinical simulation or remote teaching environments [9,10].

Several systematic reviews and meta-analyses have documented the benefits of 3D-printed models. For example, Xie et al. [11] and Ye et al. [12] reported significant improvements in medical students' learning of complex anatomical structures, both in theoretical tests and practical skills. Likewise, studies such as those by Cabrera Frías and Córdova Esparza [13] and César-Juárez et al. [14] highlight that the use of 3D printing in educational environments stimulates creative thinking, favors active learning, and increases knowledge retention.

In the particular case of neuroanatomy, these technologies have gained special relevance due to their ability to represent ascending and descending pathways and brain nuclei in a manipulable and understandable way, helping to overcome the abstraction inherent in traditional schemes [15,16]. This immersive and multisensory approach not only facilitates spatial understanding but also promotes a more interactive, meaningful learning experience aligned with the expectations of new generations of medical students [17].

This paper aimed to evaluate the impact of using 3D-printed brainstem anatomical models in teaching neuroanatomy to medical students, comparing their perceived effectiveness with the use of traditional models. This study aimed to compare medical students’ perceptions of 3D-printed brainstem anatomical models and traditional schematic diagrams used for teaching ascending and descending pathways of the central nervous system. The primary objective was to compare perceived clarity and usefulness between the two educational resources. Secondary objectives included comparing student-reported satisfaction, motivation, ease of learning, and preference for future use.

## Materials and methods

Study design

A quantitative, descriptive, cross-sectional, non-randomized, between-subjects comparative study was conducted to evaluate student perceptions of 3D-printed anatomical models versus traditional schematic diagrams in neuroanatomy teaching. The approach focused on subjective variables of satisfaction, comprehension, clarity, motivation, and perceived usefulness, using a structured questionnaire.

Population and sample

The participants were undergraduate medical students from the 3rd to 7th semester, enrolled in neuroanatomy courses during the 2025 academic year. The sample consisted of 61 students divided into two groups: a 3D-model group and a traditional-diagram group. Baseline comparability was limited to the variables collected in the study. Mean age was 20.4 ± 1.7 years in the 3D-model group and 20.8 ± 1.0 years in the traditional-diagram group. Previous exposure to 3D models and other measures of prior neuroanatomical knowledge were not collected, precluding assessment of equivalence for these potential confounders.

No a priori sample-size calculation was performed because this study was exploratory and used a convenience sample based on student availability and attendance during the educational sessions. Consequently, the study may have been underpowered to detect smaller between-group differences. Participants were not randomized, matched, or stratified. Academic performance, previous anatomy grades, baseline neuroanatomy knowledge, and previous exposure to 3D-printed anatomical models were not systematically collected; therefore, baseline equivalence between groups for these variables could not be established.

Although participants were exposed to only one educational modality during the study session, all had previously completed anatomy-related coursework and were familiar with traditional textbook diagrams. Therefore, the comparative wording of Question 10 referred to this previously familiar conventional resource and should not be interpreted as indicating exposure to both study conditions or a crossover design.

Pedagogical intervention

Both groups received instruction covering the same neuroanatomical content, specifically the ascending and descending pathways of the central nervous system. Sessions were standardized with respect to instructional content and duration, with the educational resource used during instruction constituting the principal difference between groups. The same instructor delivered both sessions; each session lasted approximately 60 minutes, using either 3D-printed anatomical models of the brainstem (intervention group) (Figure 1) or traditional textbook diagrams and two-dimensional illustrations (control group).

**Figure 1:**
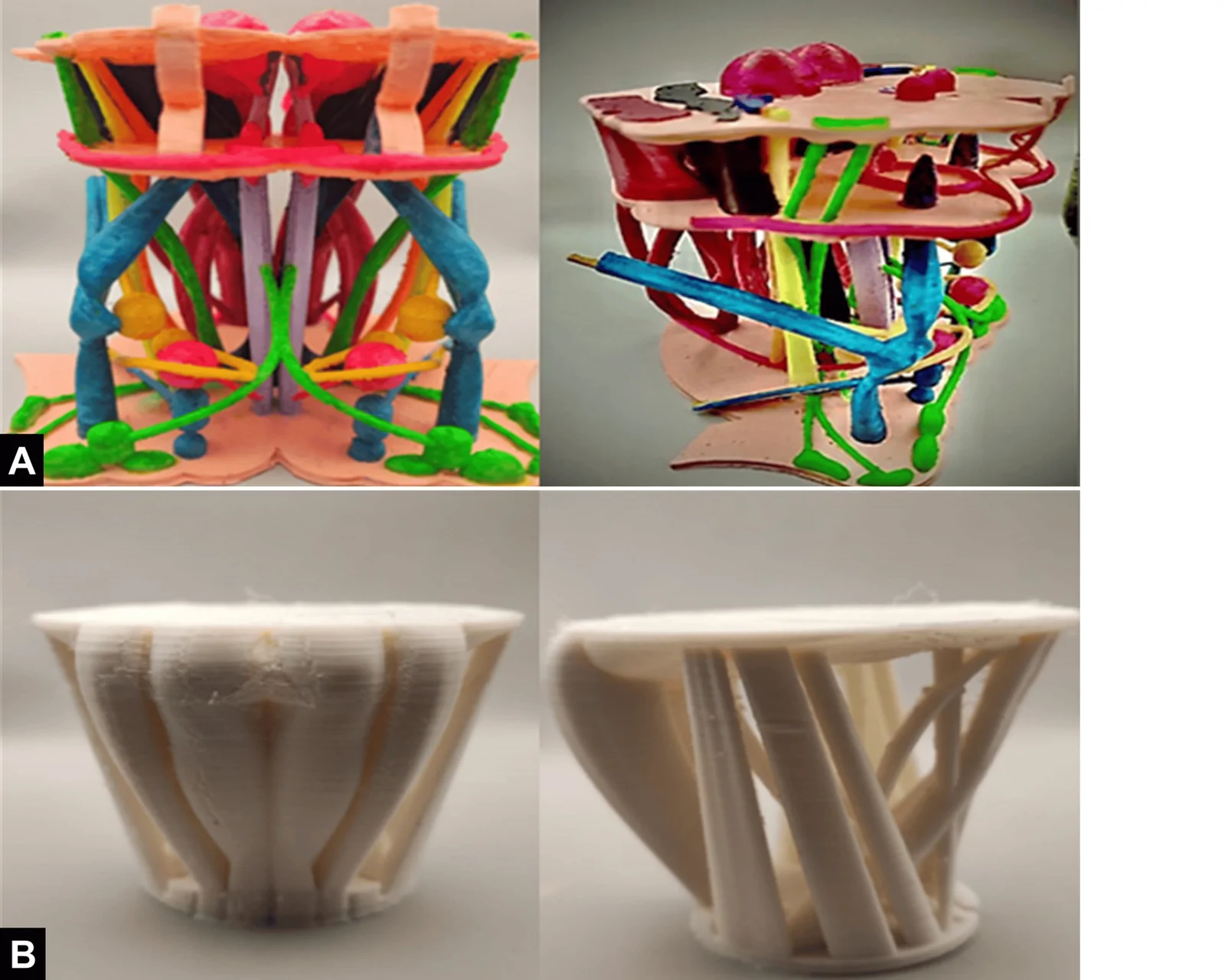
Representative 3D-printed anatomical models used during the neuroanatomy sessions. (A) Two views of the multicolored brainstem model depicting selected nuclei and ascending and descending pathways. (B) Two views of the corresponding monochromatic model, illustrating its three-dimensional structural configuration. Multiple views are provided to demonstrate the spatial relationships visible when the models are manipulated during instruction.

Evaluation instrument

Participants were assigned to two independent, non-overlapping groups according to the educational resource used during the teaching session. One group was exposed to 3D-printed anatomical models, whereas the other group was exposed to traditional schematic diagrams. Participants were exposed only to the educational modality corresponding to their group; therefore, the study followed a between-subjects rather than a crossover design. At the end of the session, participants were provided with a QR code directing them to an anonymous online survey. A structured, self-administered questionnaire was used with 11 items on a 4-point Likert scale, where 1 represented "strongly disagree/very ineffective" and 4 "strongly agree/very effective" (Table 1). The questions assessed dimensions such as perception of effectiveness, clarity in representing abstract concepts, motivation and preference, overall satisfaction, and comparison with other study materials.

**Table 1:** Questions asked in the questionnaire applied to the students participating in this study.

Question number	Question
1	Age
2	Current semester
3	On a scale of 1 to 4, do you consider textbook diagrams effective for your professional training in medicine?
4	Did the use of diagrams motivate you more?
5	Would you like to use textbook diagrams as a learning method for future topics in neuroanatomy and/or anatomy?
6	On a scale of 1 to 4, how would you rate the effectiveness of textbook diagrams in facilitating the understanding of abstract and complex concepts in neuroanatomy?
7	Do you think the use of textbook diagrams serves as a teaching tool for medical professors?
8	Does this type of tool meet your expectations as a student when compared to other teaching methods?
9	Do you think working with textbook diagrams can make learning easier and more interesting?
10	On a scale of 1 to 4, how would you rate the effectiveness of textbook diagrams compared to 3D modeling for your learning?
11	On a scale of 1 to 4, how would you rate the clarity and accuracy of the book's diagrams for illustrating the ascending and descending pathways of the spinal cord?

The questionnaire underwent qualitative content review by three experts with experience in neuroanatomy teaching. The reviewers assessed the relevance and clarity of the items before administration. No quantitative content-validity index or inter-rater agreement statistic was calculated; therefore, the instrument should be considered expert-reviewed rather than formally psychometrically validated.

All participants completed questionnaire items referring to textbook diagrams. This wording was intentional because traditional anatomical diagrams represented a previously familiar educational reference for the participating students, who had already completed anatomy-related coursework before enrollment in the study. Thus, these items were intended to capture participants’ perceptions of the conventional reference resource in the context of the educational experience rather than to constitute parallel, modality-specific versions of the questionnaire. Nevertheless, because the questionnaire items were not symmetrically worded for the two educational modalities, direct between-group interpretation of some items should be approached cautiously.

Statistical analysis

Statistical analysis was performed using IBM SPSS Statistics for Windows, Version 28 (Released 2021; IBM Corp., Armonk, New York). Descriptive statistics, including means, medians, and standard deviations, were calculated. Between-group comparisons were performed using the Mann-Whitney U test, given the ordinal nature of the 4-point Likert-scale responses and the independent-group design. Statistical significance was established at p < 0.05. Formal normality testing, including the Shapiro-Wilk test, was not performed. Additionally, effect sizes and adjustments for multiple comparisons were not calculated in the original analysis. Because the individual-level dataset was not available for the present revision, these analyses could not be performed retrospectively and are acknowledged as methodological limitations.

Ethical considerations

The study adhered to the ethical principles outlined in the Declaration of Helsinki. Participation was voluntary and anonymous. Before completing the questionnaire, participants were informed that their responses could be used for research purposes and were asked to indicate their agreement to the use of their anonymized information. Only responses from participants who provided this agreement were included in the analysis. No personally identifiable information was collected.

## Results

A total of 61 participants were included in the final analysis: 38 in the 3D-model group and 23 in the traditional-diagram group. Mean age was 20.4 ± 1.7 years and 20.8 ± 1.0 years, respectively. Regarding perceptions of the tool used, 3D-printed models obtained significantly higher scores on several key items (Figure 2).

**Figure 2:**
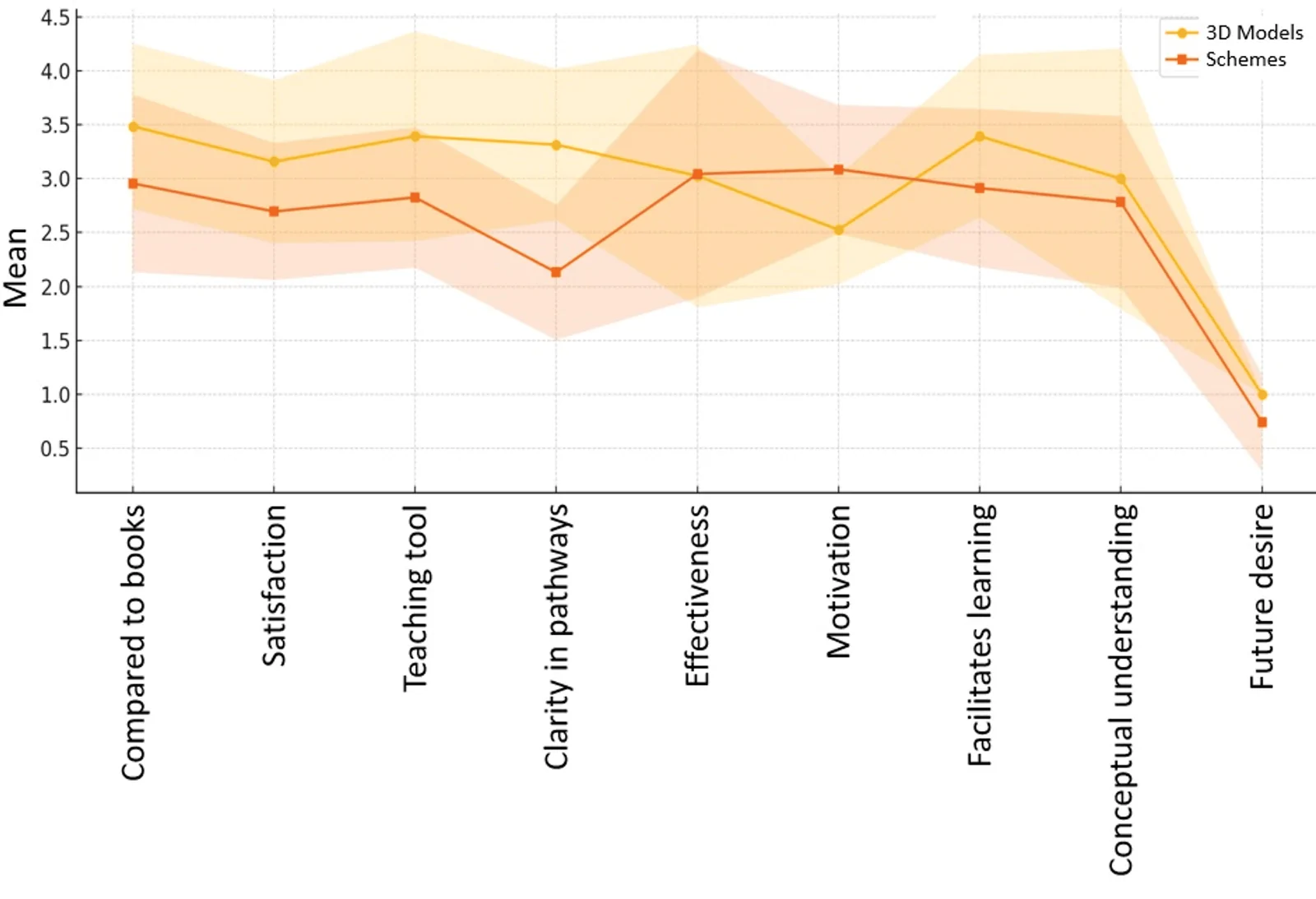
Average perception trend of students by group for each item assessed using a Likert scale (1-4). A higher overall rating of the 3D models was observed for most items, especially in satisfaction, clarity, and educational usefulness. The colored bands represent the standard deviation for each group.

The evaluation of educational outcomes demonstrated that participants rated the 3D anatomical models significantly higher than schematic diagrams in several domains. The clarity in representing abstract anatomical concepts was superior for the 3D models, with a mean score of 3.32 ± 0.70 compared with 2.13 ± 0.63 for schematic diagrams (p < 0.001). Similarly, overall satisfaction was greater among students exposed to the 3D models, achieving a mean score of 3.16 ± 0.75 versus 2.70 ± 0.63 for the schematic diagrams (p = 0.006).

Regarding the outcomes summarized in Table 2, participants in the 3D-model group reported higher ratings for the models as a useful teaching tool compared with participants in the traditional-diagram group (3.39 vs. 2.83, p = 0.0011). Similarly, the 3D-model group reported higher ratings for facilitation of learning (3.39 vs. 2.91, p = 0.0061). In contrast, motivation was higher in the traditional-diagram group than in the 3D-model group (3.09 vs. 2.53, p = 0.0007). These findings should be interpreted as exploratory between-group differences in student-reported perceptions.

**Table 2:** Available summary results for the three questionnaire outcomes retained for between-group comparison.

Item	Mean score (4-point Likert scale): 3D-printed anatomical models	Mean score (4-point Likert scale): Classic schemes	P-value
Teaching tool	3.39	2.83	0.0011
Motivation	2.53	3.09	0.0007
Facilitates learning	3.39	2.91	0.0061

In contrast, the initial motivation domain yielded an opposite trend. Traditional schematic models were perceived as more motivating than the 3D anatomical models, with mean scores of 3.09 ± 0.60 and 2.53 ± 0.51, respectively, and this difference reached statistical significance (p = 0.0007). Among the nine substantive questionnaire items, the direction and statistical significance of between-group differences varied by domain (Table 2).

## Discussion

This study compared student perceptions of the use of 3D-printed anatomical models versus traditional models in neuroanatomy teaching, revealing findings consistent with previous research on educational innovation. Specifically, 3D models were perceived as more effective tools for facilitating learning, generating greater clarity in complex concepts, and offering a more satisfying learning experience. These results align with those of Xie et al. [11] and Ye et al. [12], who reported that 3D models significantly improve performance on both theoretical and practical anatomical tests.

These findings are broadly consistent with previous literature reporting favorable educational outcomes associated with 3D anatomical models. However, unlike studies that assessed performance on theoretical or practical examinations, the present study evaluated students’ self-reported perceptions only. Therefore, our findings should not be interpreted as evidence of improved knowledge acquisition or examination performance.

The perception of greater anatomical clarity with 3D models is consistent with that described by Oliveira et al. [15] and Brumpt et al. [3], who emphasize that the tangible and spatial representation of structures facilitates the understanding of neuroanatomical pathways, especially in regions such as the brainstem, which present high functional density and complex topographic relationships. Furthermore, the finding of greater overall satisfaction and desire for future use supports the potential of these technologies as motivating and complementary elements in the medical curriculum [9,16].

However, a notable result was that the group exposed to traditional models reported greater initial motivation. This observation could be explained by the students' prior familiarity with this type of resource or by the relative novelty of the 3D model, which, although innovative, could generate some pedagogical uncertainty in first-time users [8,17]. This finding suggests that the implementation of emerging technologies should be accompanied by progressive introduction strategies and teacher training. Interestingly, participants exposed to traditional schematic diagrams reported higher motivation scores. One plausible, though untested, explanation is greater prior familiarity with conventional learning resources. However, previous exposure to either traditional diagrams or 3D-printed models was not systematically assessed; therefore, this interpretation remains speculative. Other unmeasured differences between the groups may also have contributed to this finding. Future studies should assess baseline familiarity and previous exposure to each educational modality.

Furthermore, although 3D models did not show significant differences in the perception of overall effectiveness or conceptual understanding compared to diagrams, the scores obtained were similar or higher, which is consistent with studies indicating that the main benefit of these tools lies in the improvement of the visual and manipulative experience, rather than the absolute replacement of previous methods [5,10].

This provides empirical evidence in support of integrating 3D-printed models into the instructional design of morphofunctional subjects. This proposal aligns with the global movement toward didactic innovation in medical education, especially in settings where the availability of cadaveric material is limited [13,14].

Limitations

Several limitations should be considered when interpreting these findings. First, this was a small, non-randomized, between-subjects study based on convenience sampling, with unequal group sizes, which limits generalizability and may introduce selection bias. Participants were not matched or stratified, and baseline academic performance, prior anatomy grades, baseline neuroanatomy knowledge, and previous exposure to 3D-printed anatomical models were not systematically assessed. Second, although the questionnaire underwent expert review, quantitative measures of content validity or psychometric reliability were not obtained. Several questionnaire items were also framed specifically around textbook diagrams, which may limit construct validity for direct between-group comparisons. Third, the statistical analysis did not include adjustment for multiple comparisons or effect-size estimates; therefore, the reported p-values should be interpreted as exploratory. Finally, outcomes were based on immediate self-reported perceptions rather than objective measures of knowledge acquisition, examination performance, long-term retention, transfer of learning, or clinical application.

## Conclusions

In this exploratory study, medical students exposed to 3D-printed neuroanatomical models reported favorable perceptions regarding their usefulness as a teaching tool and their ability to facilitate learning, whereas students exposed to traditional schematic diagrams reported higher motivation scores. These findings suggest that 3D-printed models may represent a useful complementary resource for neuroanatomy education rather than a replacement for conventional teaching materials.

Given the non-randomized between-subjects design, convenience sampling, small and unequal group sizes, questionnaire limitations, and reliance on immediate self-reported perceptions, the present findings should not be interpreted as evidence that 3D-printed models improve objective learning outcomes. Larger randomized and longitudinal studies incorporating validated instruments and objective measures of knowledge acquisition, retention, and academic performance are needed to determine their educational effectiveness.
